# Immune-Related Adverse Events of Genitourinary Cancer Patients, a Retrospective Cohort Study

**DOI:** 10.3390/cancers16173045

**Published:** 2024-08-31

**Authors:** John C. Hunting, Logan Deyo, Eric Olson, Andrew T. Faucheux, Sarah N. Price, Thomas W. Lycan

**Affiliations:** 1 Department of Internal Medicine, Wake Forest School of Medicine, Wake Forest University, Winston-Salem, NC 27157, USA; 2 Department of Hematology & Oncology, Wake Forest School of Medicine, Wake Forest University, Winston-Salem, NC 27157, USA; 3 Department of Social Sciences and Health Policy, Wake Forest School of Medicine, Wake Forest University, Winston-Salem, NC 27157, USA; sarahpr@wakehealth.edu

**Keywords:** immunotherapy, genitourinary cancer, adverse events, outcomes, and survival

## Abstract

**Simple Summary:**

Immune checkpoint inhibitors (ICIs) are a common and growing type of cancer treatment, but they can cause side effects. This study aims to understand these side effects in patients with kidney cancer and bladder cancer. We compared the types and severity of side effects between these two groups to determine if there are differences. We also compared them to other tumor types to see if there is a unique pattern. The findings will help doctors better predict and manage side effects. This will hopefully lead to improving patient care for those receiving ICI treatment.

**Abstract:**

Background: Immune checkpoint inhibitors (ICIs) have become common lines of therapy for genitourinary cancers (GUcs). Given their widespread use, understanding the risk factors, comparative profiles, and timing of immune-related adverse events (irAEs) is essential. Methods: We created an IRB-approved retrospective registry of all patients who received at least one dose of an ICI for any indication between 1 February 2011 and 7 April 2022 at a comprehensive cancer center and its outreach clinics. Dichotomous outcomes were modeled using multivariable logistic regression. Survival outcomes were compared using multivariable Cox regression. Results: Among 3101 patients, 196 had renal cell carcinoma (RCC) and 170 had urothelial tumors. RCC patients were more likely to experience irAEs (OR 1.78; 95% CI 1.32–2.39), whereas urothelial carcinoma patients were not (OR 1.22; 95% CI 0.88–1.67). RCC patients were more prone to dermatitis, thyroiditis, acute kidney injury, and myocarditis, compared to other tumors, while urothelial carcinoma patients were not. The impact of irAEs on survival was not significantly different for GUcs compared to other tumors. Conclusions: RCC primaries have a significantly different irAE profile than most tumors, as opposed to urothelial primaries. Further, RCC was more likely to experience any irAEs. Heterogeneity of survival benefits by irAEs was not seen.

## 1. Introduction

Over the last decade, immunotherapy has been at the forefront of cancer research across most solid cancer types and has become increasingly efficacious, with favorable outcomes as early-line therapy for genitourinary cancers (GUcs). For GUcs, currently approved immunotherapies include immune checkpoint inhibitors (ICIs) for metastatic/advanced renal cell carcinoma (RCC) and urothelial/bladder cancers [1,2,3,4,5,6,7,8], recombinant interleukin-2 for metastatic RCC, and the sipuleucel-t vaccine for metastatic castration-resistant prostate cancer [1,2,3,4,5,6,7,8,9,10]. ICIs, which include anticytotoxic T-lymphocyte antigen 4 (CTLA-4), antiprogrammed cell death protein 1 (PD-1), and antiprogrammed death-ligand 1 (PD-L1) monoclonal antibodies, have especially exhibited advantageous outcome results for GUcs when combined with other agents, such as tyrosine-kinase inhibitors (TKI). Landmark trials, including KEYNOTE-426, JAVELIN 101, IMmotion 151, Checkmate 214, and Checkmate 9ER, have featured various ICIs in conjunction with a TKI and have shown superior survival benefit over prior first-line monotherapy TKI sunitinib for metastatic RCC [11,12,13,14,15].

A growing body of research supports ICI monotherapy, including KEYNOTE-057 with FDA-approval for pembrolizumab monotherapy in high-risk non-muscle invasive bladder cancer unresponsive to first-line bacillus Calmette–Guérin (BCG) [16]. Checkmate274 showed a disease-free survival (DFS) benefit with adjuvant nivolumab versus placebo in muscle-invasive urothelial carcinoma after radical cystectomy [17]. Finally, KEYNOTE 564 demonstrated a recurrence-free survival (RFS) benefit of adjuvant pembrolizumab in patients with high-risk localized/locally advanced kidney cancer [18]. Unlike renal cell carcinoma and urothelial/bladder cancer, prostate cancer has not exhibited promising response to ICIs, likely due to the “bland” tumor microenvironment of the prostate with low mutational burden and lack of adequate inflammatory penetrance [19]. Overall, these treatment modalities have become important components of the treatment paradigm for GU cancer. However, despite ICIs being generally more tolerable than other therapies, their expanding indications also increase the incidence of immune-related adverse events (irAEs).

“Immune-related adverse events”, or irAEs, have presented in clinical practice in a varying degree of illness types and severities, whether mild and limiting in nature or severe, leading to hospitalization and/or death. These irAEs are often a diagnosis of exclusion and, thus, can be challenging to identify and manage. Depending on their severity, irAEs can also halt further treatment of the patient’s cancer, leading to a delay in care.

Regarding the patterns and incidence of irAEs based on tumor type and ICI class (anti-CTLA-4, anti-PD-1, and anti-PD-L1 monoclonal antibodies), studies show that the most commonly reported events that occur are endocrine (thyroid disorders such as hypothyroidism and hyperthyroidism followed by pituitary and adrenal dysfunction), gastrointestinal (diarrhea, colitis, nausea), lung (pneumonitis), skin (rash, pruritus, and vitiligo), and musculoskeletal (arthralgia and myalgia) [20,21,22]. Grade ≥ 3, or more severe, irAEs appear to be more common with anti-CTLA-4 compared with anti-PD-1 ICIs, up to 31% versus 10% with OR 4.0 in one study [20]. Interestingly though, it has been posited that the development and severity of irAEs may correlate with tumor response. Several studies evaluating different ICIs have shown an association between the development of irAEs and long-term survival, particularly in patients with melanoma, non-small-cell lung cancer (NSCLC), and urothelial cancers; however, the mechanism remains unknown and needs to be further explored [23,24,25,26,27,28].

Regarding irAEs in GU cancers, one specific systematic review and meta-analysis of GU clinical trials (over 92 studies on renal, urothelial/bladder, prostate, adrenal, testicular, and penile cancers) and associated irAEs yielded a pooled overall incidence of 34.3% for any-grade irAEs and 10.2% for grade ≥ 3 irAEs. The renal cell cancer group overall had a higher incidence of any-grade irAEs (42.7%) than the urothelial group (24.9%). The pooled incidence for any-grade irAEs was also higher for dual ICI therapy (anti–PD-1 or anti–PD-L1 plus anti–CTLA-4) (78.0%) and for ICIs combined with a targeted therapy (TKIs) (48.8%) as compared to single ICI agent therapy (24.9%) [29]. Overall, a fair number of patients experience irAEs that will typically resolve after discontinuation and the use of a prolonged high-dose corticosteroid taper.

Rechallenging with the same agent after a grade ≥ 3 event is strongly discouraged; however, resuming treatment after milder irAE cases has been studied for irAE recurrence. In a cohort study of 24,079 irAEs associated with at least one ICI, the recurrence rate of the same event that prompted discontinuation of ICI therapy was 28.8% after rechallenge with the same ICI [30]. Regarding specific irAEs in this study, colitis, hepatitis, and pneumonitis had higher recurrence rates compared with other events.

Immune-related adverse events are routinely accounted for in individual trials and studies; however, there is an overall lack of literature focused solely on irAE profiles in routine clinical practice. Understanding the relative incidence of irAEs according to solid tumor type and the resultant immune-related side effect profile can further inform the medical community for risk/benefit discussions when starting ICIs and when to promptly recognize and treat these complications. This reality is especially important for GU cancers, as ICIs have become increasingly more popular options for treatment, which, in addition to other agents such as TKIs, have been shown to synergistically increase the risk of irAEs.

This project aimed to compare patients with GU tumors to the broader population of patients treated with ICIs in the following factors: (1) demographic and clinical characteristics, (2) odds of experiencing any irAEs, (3) types of irAEs experienced, (4) timing of first irAE, and (5) impact of irAEs on survival. We aim to further support the prior literature that GU cancers (RCC and urothelial/bladder) have a significantly different irAE profile than other cancers, and that the influence of irAEs on survival (a suspected benefit) is heterogenous for GU cancers when compared to other cancers.

## 2. Methods

### 2.1. Data Acquisition

The present study was approved by the institutional review board at Wake Forest School of Medicine. Informed consent was waived by the IRB due to the retrospective nature of the data, which were collected as part of standard care. Patients were eligible for this study if they were at least 18 years older and were diagnosed with genitourinary cancer, including the renal or urothelial tract. Further, they were required to have received at least one dose of an ICI between 1 February 2011 and 7 April 2022 at either a Comprehensive Cancer Center or any of its associated outreach clinics. Patients with prior malignancies were allowed to remain in this study as a pragmatic consideration. irAE subtypes by tumor primary excluding prior malignancies are included as a Supplementary Table S1. Study personnel from Vasta Global, LLC manually abstracted data on treatment-emergent adverse events through review of patients’ electronic health records (EHRs). A secure, cloud-based registry (REDCap) was used for data storage. Trained clinical research specialists then validated the abstracted data using data quality rules and resolved all discrepancies.

### 2.2. Definition of irAE

The primary exposure of irAEs was determined by retrospective review of patients’ problem lists, clinic notes, and hospitalization notes. Given the nature of irAE diagnosis of exclusion, each irAE was diagnosed by the responsible physician at the time of care. All types of irAEs were included.

### 2.3. Definition of Adverse Event Grade

Adverse event grading was defined using the Common Terminology Criteria for Adverse Events (CTCAE) supported by the National Cancer Institute (NCI) [31]. Low-grade irAE was defined as either grade 1 or 2. High-grade irAE was defined as either grade 3 or 4.

### 2.4. Censoring of Time Data

Patients who lacked explicit time of irAE, progression, or survival data were censored at the time of their last follow-up. This last follow-up encompassed any clinic visit, lab draw, or other encounter that confirmed the patient’s survival up to that specific point. The time of the last follow-up was utilized to censor any time variable (time of irAE, progression-free survival, or overall survival) if these data were not previously recorded.

### 2.5. Statistics 

All analyses were conducted using SAS version 9.4 (Raleigh, NC, USA). Descriptive statistics were calculated and reported for all relevant variables. Continuous variables were reported using either mean and standard deviation or median and interquartile range depending on distribution. Categorical variables were reported using count and frequency. Univariate comparisons were made between continuous variables with either two-sample T-test or Kruskal–Wallis test, depending on statistical assumptions. Categorical variables were compared using either chi-square or Fisher’s exact test, depending on applicable assumptions. Dichotomous outcomes were modeled using multivariable logistic regression. Survival data were compared using Cox proportional hazards models. Potential confounders were considered for inclusion if associated with the tumor primary, as described in univariate analysis. Model selection was performed among these variables in a stepwise manner. Model entry criteria was set at *p* ≤ 0.2 with model retention criteria set at *p* ≤ 0.1. Significance was defined as *p* < 0.05. Multiple testing correction was not performed.

## 3. Results

### 3.1. Patient Characteristics by Tumor Type

The cohort included 3101 patients; among them, 196 (6.3%) were renal cell carcinoma (RCC) while 170 (5.5%) were urothelial tumor primaries. Mean age at ICI initiation was 63 years old for RCC and 69 years old for urothelial carcinoma, compared to the rest of the cohort at 63 years old (*p* < 0.001). The GU cancers were predominately male (76% and 74% RCC and urothelial, respectively) and Caucasian (89% and 88% RCC and urothelial, respectively). Smoking history was significantly heterogenous (*p* < 0.001) when compared across all groups. RCC patients had the highest proportion of never smoking (41%), while urothelial tumor primaries were the least likely to have never smoked (18%), as fully described in Table 1.

Among comorbidities, both RCC and urothelial primaries were more likely to have GI (diarrhea/constipation), vertebral disk degeneration, cardiovascular disease, visual impairment, diabetes mellitus, and hearing impairment, as shown in Table 2. Significant variation was also seen among COPD, with RCC being significantly lower in prevalence (24% among RCC vs. 41% overall; *p* < 0.001). RCC patients were also more likely to have thyroid dysfunction (24% vs. 17% overall; *p* = 0.02). Patients with urothelial carcinoma were significantly more likely to have had a prior malignancy when compared to RCC (28% vs. 18%, respectively; *p* = 0.008).

### 3.2. Immune-Related Adverse Events by Tumor Type

Among the entire cohort, 1169 (37.7%) patients experienced any irAEs. Among respective tumor primary, RCC had 101 (51.5%) irAEs, while urothelial had 72 (42.4%), which was significantly heterogenous from the other tumor types (36%) (*p* < 0.001). High-grade events occurred in 41 (20.9%) RCC patients and 23 (13.5%) urothelial patients, which, again, was significantly different from the other tumor primaries (13.5%) (*p* = 0.014). The most common irAEs overall included dermatitis (8.6%), thyroiditis (6.7%), pneumonitis (6.1%), and colitis (6.0%) as shown in Table 3. Notably, in the majority of event types, RCC was meaningfully higher than the total population percentage, with significant elevations in dermatitis, thyroiditis, acute kidney injury, hypophysitis, pancreatitis, central nervous system involvement, and cardiovascular. Urothelial carcinoma was more consistent with the other tumor primaries in the overall profile of irAEs, though it also had an increased prevalence of acute kidney injury.

To assess the odds of developing an irAE by tumor primary, a multivariable logistic regression model was built. Controlling for BMI, sex, age, and race, tumor primary remained significantly associated with the odds of developing an irAE. With “other” as reference group, RCC had an increased odds of developing an irAE: (OR 1.78|95% CI 1.32–2.39) but urothelial carcinoma did not (OR 1.22|95% CI 0.88–1.67).

**Figure 1:**
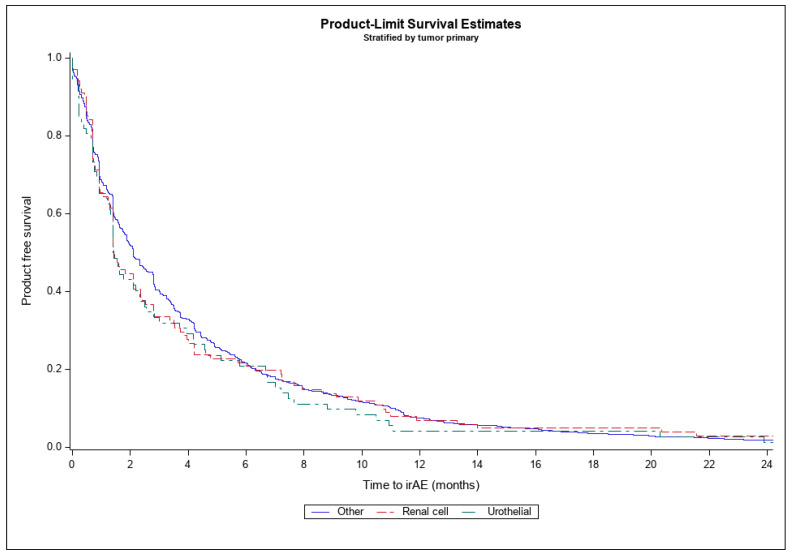
Time to irAE by tumor primary.

Time to first irAE, described in Table 4, was initially compared between tumor primaries (RCC, urothelial carcinoma, and other) via the Kaplan–Meier method, shown in Figure 1. At baseline, there was no significant difference between groups (all pairwise comparison *p*-values > 0.05). A multivariable Cox regression model was built to assess the influence of tumor primary on time to irAE.

Multivariable Cox regression models were built to assess the influence of irAEs by tumor primary on both progression-free and overall survival. Primary tumor, any grade irAE, age, and prior malignancy were included in both final models based on a priori consideration and stepwise selection. Prior rheumatological comorbidity was also included in the PFS model based on model selection parameters. While accounting for the other variables, irAE was significantly associated with improved PFS and OS. To explore whether there was heterogeneity in the impact of irAE on progression-free survival, an interaction term was tested and was not significant (*p* = 0.858). Additionally, the potential for heterogeneity regarding overall survival was tested with an interaction term between tumor primary and irAE and was not significant (*p* = 0.105).

## 4. Discussion

This study provides a comprehensive analysis of immune-related adverse events (irAEs) in genitourinary (GU) cancers, specifically renal cell carcinoma (RCC) and urothelial carcinoma, within a large cohort of patients receiving immune checkpoint inhibitors (ICIs). Our findings highlight several key insights into the incidence, subtype distribution, and potential underlying mechanisms of irAEs in these patient populations. As is consistent with the prior literature, there was, again, a survival benefit to a patient experiencing an irAE; however, there was no significant evidence that the survival benefit for GU cancers was significantly different than other tumor primaries [23,32].

At baseline, the GU cancer population was significantly and meaningfully different than the rest of the population receiving immunotherapy. Consistent with the prior literature, both RCC and urothelial carcinoma patients are more commonly male [33,34]. RCC showed a much higher proportion of patients who had never smoked, at nearly 41%, though over 80% of the urothelial carcinoma patients had previously or currently smoked, which aligns with its known risk factors. Along these lines, it is understandable that the prevalence of COPD was significantly lower among the RCC population versus both urothelial carcinoma and the rest of the population, around 40-42%. There was, further, a number of differences in comorbidities between tumor primaries connecting both demographics and potential risk factors for subtypes of irAEs. Among RCC and urothelial carcinoma, compared to other tumors, metabolic comorbidities were more common, including cardiovascular disease (43% vs. 41% vs. 36%, respectively) and diabetes (34% vs. 31% vs. 25%, respectively. This coincides with their respective increased incidence of smoking as well, compared to other tumor types (*p* < 0.001).

Overall, irAEs were more common among RCC patients when compared to the non-GU cancer immunotherapy population (OR 1.78|95% CI 1.32–2.39). Though there may be multiple mechanisms including both comorbidities and tumor properties, it is also likely that the common use of dual ICI ipilimumab and nivolumab has an impact, consistent with the previously discussed literature [29]. It is important to note, though, as noted in the limitations, that this study did not contain data on specific ICIs used. Though urothelial carcinoma also had a positive point value with an odds ratio of 1.22, it was not significant (95% CI 0.88–1.67). Single-agent pembrolizumab is the most commonly used as the immunotherapy agent for bladder cancer [16].

Considering the driving mechanism for the increased incidence of irAEs among RCC, there remain the two theories of hidden autoimmunity and molecular mimicry. Notably, when considering hidden autoimmunity, there was a significant elevation in rheumatological comorbidities among tumor types largely driven by urothelial carcinoma compared to other tumor primaries (48% vs. 37%). However, RCC and other tumors were clinically comparable (38% vs. 37%); thus, this does not support the overall increased OR noted above. Molecular mimicry then becomes an interesting mechanism that could be supported when comparing the specific irAE types.

The subtype analysis revealed distinct irAE profiles between RCC and urothelial carcinoma patients. Dermatitis was the most common event among the population as a whole (8%), though it was twice as common among RCC patients (16%; *p* < 0.001). Further, thyroiditis was nearly twice as common among RCC patients than the other tumor primaries (11% vs. 6%; *p* = 0.006). RCC irAEs were further significantly elevated in hepatitis, hypophysitis, diabetes, myositis, acute kidney injury (AKI), central nervous system, cardiovascular, and infusion reactions, as described in Table 3. Noticeably, these target organs are all histologically distinct, and though some are anatomically proximal (AKI, hepatitis, diabetes), the others are not. There were no subtypes of irAE in which RCC was less likely to experience an irAE. In comparison to other tumors, urothelial carcinoma was not significantly different from the rest of the tumor population. One consideration in this difference could be metastatic patterns. RCC often metastasizes hematological spread, being one of the most common tumors to metastasize to the brain [35]. Prior studies have shown that tumors that commonly metastasize to the CNS are more likely to develop CNS irAEs [20,36]. Though RCC did not show an increased odds of pneumonitis (lung being a common metastatic site), the “other” category included lung cancer; thus, it is unlikely that RCC would overcome the influence of this native tissue and molecular mimicry. By contrast, urothelial typically metastasizes locally. If subclinical metastases predispose an organ to irAEs, this could be a possible mechanism.

Considered influencing elements, again, are the possible synergistic risk of dual ICIs, whereas most other tumors treated with ICIs use monotherapy. Further, though, it remains possible that tumor properties and the possibility of subclinical systemic involvement/metastases would then predispose to irAEs. Though RCC did not show increased odds of pneumonitis (with lung being a common metastatic site), it was significantly more likely to involve hepatitis and central nervous system involvement. A prior study has shown that tumor primaries known to favor brain metastases (lung, RCC, melanoma) are more likely to involve CNS sites of irAEs [20]. However, it remains unclear why each organ may be involved while others are not, thus making it difficult to predict which organ may be involved, let alone predict the events at all.

This study is not without its limitations. First, the population is from a single institution and, thus, generalizability must be considered for a broader population. Next, given that irAE remains a diagnosis of exclusion, there is a risk of variability and subjectivity between detection rates at both an institutional level and provider level. Finally, the data used for this analysis did not contain detailed data on the specific ICIs used. It is possible and likely that there are drug specific impacts on irAE profiles that should be explored in the future.

Further research is warranted to dissect the heterogeneity of irAEs, particularly in relation to specific tumor types and treatment regimens. Understanding the molecular and immunological basis of irAE development could lead to more personalized approaches to ICI therapy, minimizing adverse events while maximizing therapeutic efficacy. Additionally, prospective studies that incorporate comprehensive immune profiling and biomarker analysis could provide deeper insights into the risk factors and potential interventions for irAEs in GU cancers.

## 5. Conclusions

Immune-related adverse events remain a crucial and elusive element of treating genitourinary cancers with immunotherapy. While urothelial carcinoma seems to more closely resemble other tumor primaries irAE profile, RCC is uniquely poised to experience more events in a wide array of organ systems.

## Figures and Tables

**Table 1:** Demographics by tumor primary.

	Renal Cell (*n* = 196)	Urothelial (*n* = 170)	Other (*n* = 2735)	Total (*N* = 3101)	*p* Value
**Age**	62.51 (11.01)	68.48 (11.99)	63.16 (11.77)	63.41 (11.80)	<0.001 ^1^
**Female**	47 (23.98%)	44 (25.88%)	1145 (41.86%)	1236 (39.86%)	<0.001 ^2^
**Race**					0.036 ^2^
Black/African American	11 (5.61%)	16 (9.41%)	333 (12.18%)	360 (11.61%)	
Other	9 (4.59%)	4 (2.35%)	78 (2.85%)	91 (2.93%)	
White/Caucasian	176 (89.80%)	150 (88.24%)	2324 (84.97%)	2650 (85.46%)	
**Smoking History**					<0.001 ^2^
Current	25 (12.76%)	30 (17.65%)	621 (22.71%)	676 (21.80%)	
Former	90 (45.92%)	109 (64.12%)	1425 (52.10%)	1624 (52.37%)	
Never	81 (41.33%)	31 (18.24%)	689 (25.19%)	801 (25.83%)	
**Tumor Primary**					
Head/neck	na	na	253 (9.25%)	na	
Melanoma	na	na	444 (16.23%)	na	
NSCLC	na	na	1114 (40.73%)	na	
Other	na	na	924 (33.78%)	na	
**Disease Stage**					<0.001 ^2^
1	20 (12.12%)	18 (14.40%)	217 (9.02%)	255 (9.45%)	
2	22 (13.33%)	38 (30.40%)	284 (11.80%)	344 (12.75%)	
3	37 (22.42%)	38 (30.40%)	678 (28.17%)	753 (27.92%)	
4	86 (52.12%)	31 (24.80%)	1228 (51.02%)	1345 (49.87%)	
Missing	31 (.%)	45 (.%)	328 (.%)	404	
**Immunotherapy line of treatment**					<0.001 ^2^
1	102 (52.04%)	49 (28.82%)	1281 (46.84%)	1432 (46.18%)	
2	61 (31.12%)	82 (48.24%)	976 (35.69%)	1119 (36.09%)	
3	17 (8.67%)	31 (18.24%)	280 (10.24%)	328 (10.58%)	
4	16 (8.16%)	8 (4.71%)	198 (7.24%)	222 (7.16%)	

^1^ Kruskal–Wallis; ^2^ chi-square; patient race self-reported; age reported in years at initiation of ICI. na: Not Available.

**Table 2:** Comorbidities by tumor primary.

Comorbidity	Renal Cell (*n* = 196)	Urothelial (*n* = 170)	Other (*n* = 2735)	Total (*N* = 3101)	*p* Value
**Gastrointestinal**	104 (53.06%)	94 (55.29%)	1381 (50.49%)	1579 (50.92%)	0.397 ^1^
**Psychological**	80 (40.82%)	63 (37.06%)	1236 (45.19%)	1379 (44.47%)	0.067 ^1^
**COPD**	48 (24.49%)	68 (40.00%)	1166 (42.63%)	1282 (41.34%)	<0.001 ^1^
**Disk degeneration**	112 (57.14%)	119 (70.00%)	1036 (37.88%)	1267 (40.86%)	<0.001 ^1^
**Rheumatological**	74 (37.76%)	82 (48.24%)	1043 (38.14%)	1199 (38.66%)	0.032 ^1^
**Cardiovascular** **disease**	85 (43.37%)	70 (41.18%)	981 (35.87%)	1136 (36.63%)	0.049 ^1^
**Visual impairment**	59 (30.10%)	58 (34.12%)	716 (26.18%)	833 (26.86%)	0.045 ^1^
**Diabetes mellitus**	66 (33.67%)	53 (31.18%)	695 (25.41%)	814 (26.25%)	0.013 ^1^
**Prior malignancy**	31 (15.82%)	48 (28.24%)	673 (24.61%)	752 (24.25%)	0.008 ^1^
**Thyroid** **dysfunction**	48 (24.49%)	25 (14.71%)	462 (16.89%)	535 (17.25%)	0.020 ^1^
**Hearing** **impairment**	24 (12.24%)	32 (18.82%)	304 (11.12%)	360 (11.61%)	0.013 ^1^
**Asthma**	23 (11.73%)	19 (11.18%)	308 (11.26%)	350 (11.29%)	0.973 ^1^
**Neurological**	19 (9.69%)	14 (8.24%)	289 (10.57%)	322 (10.38%)	0.658 ^1^
**Osteoporosis**	8 (4.08%)	9 (5.29%)	167 (6.11%)	184 (5.93%)	0.540 ^1^
**HIV**	0 (0.00%)	2 (1.18%)	16 (0.59%)	18 (0.58%)	0.267 ^1^
**Irritable bowel disease**	2 (1.02%)	1 (0.59%)	19 (0.69%)	22 (0.71%)	0.770 ^1^

^1^ Fisher exact; gastrointestinal includes ulcerative disease, hernia, gastric reflux; rheumatological includes arthritis, connective tissue, or other rheumatic disease; neurological disease includes CVA, TIA, dementia, hemiplegia; psychological includes history of inpatient psychiatry consult or history of psychiatric medication; thyroid dysfunction includes both hypothyroidism and hyperthyroidism; visual impairment includes cataracts, glaucoma, macular degeneration, etc.; hearing impairment includes prior documentation of difficulty hearing or use of hearing aids.

**Table 3:** irAEs by tumor primary.

	Renal Cell (*N* = 196)	Urothelial (*N* = 170)	Other (*N* = 2735)	*p*-Values	
				RCC vs. Other	Urothelial vs. Other
**Dermatitis**	32 (16.33%)	17 (10.00%)	218 (7.97%)	<0.001 ^1^	0.346 ^1^
**Thyroiditis**	22 (11.22%)	16 (9.41%)	169 (6.18%)	0.006 ^1^	0.104 ^2^
**Colitis**	18 (9.18%)	10 (5.88%)	158 (5.78%)	0.052 ^1^	0.954 ^1^
**Pneumonitis**	11 (5.61%)	8 (4.71%)	170 (6.22%)	0.735 ^1^	0.512 ^2^
**Hepatitis**	9 (4.59%)	2 (1.18%)	53 (1.94%)	0.013 ^1^	0.769 ^2^
**Acute kidney injury**	7 (3.57%)	4 (2.35%)	34 (1.24%)	0.007 ^1^	0.279 ^2^
**Hypophysitis**	6 (3.06%)	1 (0.59%)	20 (0.73%)	0.001 ^1^	1.000 ^2^
**Pancreatitis/DM**	5 (2.55%)	0 (0.00%)	7 (0.26%)	<0.001 ^1^	0.999 ^2^
**Arthritis**	4 (2.04%)	4 (2.35%)	61 (2.23%)	0.999 ^2^	0.790 ^2^
**Myositis**	4 (2.04%)	1 (0.59%)	16 (0.59%)	0.040 ^2^	0.999 ^2^
**Central nervous system**	3 (1.53%)	0 (0.00%)	4 (0.15%)	0.008 ^2^	0.999 ^2^
**Peripheral nervous system**	3 (1.53%)	0 (0.00%)	13 (0.48%)	0.087 ^2^	0.999 ^2^
**Cardiovascular**	3 (1.53%)	2 (1.18%)	8 (0.29%)	0.500 ^2^	0.112 ^2^
**Hematological**	1 (0.51%)	0 (0.00%)	9 (0.33%)	0.033 ^2^	0.999 ^2^
**Infusion reaction**	4 (2.04%)	3 (1.76%)	20 (0.73%)	0.072 ^2^	0.148 ^2^

^1^ Chi-square; ^2^ Fisher exact; each column percentages within that group; irAE = immune-related adverse event.

**Table 4:** Time to irAE by tumor primary.

	Renal Cell (*n* = 196)	Urothelial (*n* = 170)	Other (*n* = 2735)
**Time to irAE**	4.5 (8.0)	3.6 (5.1)	4.5 (7.7)

All variables reported as mean (std) in months.

## Data Availability

Data will be made available per request to the corresponding author.

## Supplementary Materials

The following are available online at https://www.mdpi.com/article/10.3390/cancers16173045/s1, Table S1: irAE type by GU tumor (excluding prior malignancy).
