# Supplementary material for: Immune-Related Adverse Events of Genitourinary Cancer Patients, a Retrospective Cohort Study
Source: Cancers (Basel). 2024 Aug 31;16(17):3045. doi: 10.3390/cancers16173045 (PMC11394475; doi:10.3390/cancers16173045)
Supplement: Supplementary file 1 [file cancers-16-03045-s001.zip › cancers-3133131-supplementary.pdf]

Table S1: irAE type by GU tumor (excluding prior malignancy)

	Renal cell (n = 165)	Urothelial (n = 122)	Other (n = 2062)	***P*-values**	
				RCC vs other	Urothelial vs other
**Dermatitis**	28 (16.97%)	13 (10.66%)	167 (8.10%)	<0.001[1]	0.318[1]
**Thyroiditis**	17 (10.30%)	13 (10.66%)	138 (6.69%)	0.079[1]	0.098[2]
**Colitis**	15 (9.09%)	6 (4.92%)	119 (5.77%)	0.084[1]	0.693[1]
**Pneumonitis**	8 (4.85%)	6 (4.92%)	131 (6.35%)	0.442[1]	0.700[2]
**Hepatitis**	8 (4.85%)	2 (1.64%)	45 (2.18%)	0.031[1]	0.999[2]
**Acute Kidney Injury**	7 (4.24%)	3 (2.46%)	23 (1.12%)	0.005[2]	0.174[2]
**Hypophysitis**	1 (0.61%)	1 (0.82%)	13 (0.63%)	0.970[1]	0.554[2]
**Pancreatitis/DM**	5 (3.03%)	0 (0.00%)	5 (0.24%)	<0.001[1]	0.999[2]
**Arthritis**	3 (1.82%)	3 (2.46%)	41 (1.99%)	1.000[2]	0.734[2]
**Myositis**	3 (1.82%)	0 (0.00%)	9 (0.44%)	0.053[2]	0.999[2]
**Central nervous system**	3 (1.82%)	0 (0.00%)	4 (0.19%)	0.011[2]	0.999[2]
**Peripheral nervous system**	3 (1.82%)	0 (0.00%)	9 (0.44%)	0.053[2]	0.999[2]
**Cardiovascular**	3 (1.82%)	1 (0.82%)	5 (0.24%)	0.017[2]	0.292[2]
**Hematological**	1 (0.61%)	0 (0.00%)	8 (0.39%)	0.500[2]	0.999[2]
**Infusion reaction**	3 (1.82%)	2 (1.64%)	18 (0.87%)	0.200[2]	0.309[2]

[1]Chi-Square; [2]Fisher Exact; Each column percentages within that group.; irAE = immune related adverse event.
